# Clinical trials in dementia with Lewy bodies: the evolving concept of co-pathologies, patient selection and biomarkers

**DOI:** 10.1097/WCO.0000000000001173

**Published:** 2023-06-09

**Authors:** Lucy L. Gibson, Carla Abdelnour, Joyce Chong, Clive Ballard, Dag Aarsland

**Affiliations:** a Old Age Psychiatry Department, Institute of Psychiatry, Psychology and Neuroscience, King's College London, London, UK; b Department of Neurology and Neurological Sciences, Stanford University School of Medicine, Stanford, California, USA; c Medical School (HB), University of Exeter, Exeter, UK; d Centre for Age-Related Medicine, Stavanger University Hospital, Stavanger, Norway

**Keywords:** biomarkers, clinical trials, dementia with Lewy bodies

## Abstract

Currently, no disease modifying therapies (DMTs) have been approved for use in dementia with Lewy bodies (DLB). Clinical trials face difficulties due to the clinical and neuropathological heterogeneity of the condition with a diverse array of neuropathogenic mechanisms contributing to the clinical phenotype. The purpose of this review is to describe how recent advances in the development of biofluid biomarkers may be used in clinical trials to tackle some of these challenges.

**Recent findings:**

Biomarkers are essential both to support the accurate diagnosis of DLB and to delineate the influence of coexisting pathologies. Recent advances in the development of α-synuclein seeding amplification assays (SAA) allow accurate identification of α-synuclein from the prodromal stages in DLB. Additionally, validation of plasma phosphorylated tau assays in DLB is ongoing and offers an accessible biomarker to indicate the existence of AD co-pathology. Use of biomarkers for diagnosis and group stratification in clinical trials of DLB is growing and likely to be of increasing importance in the future.

**Summary:**

*In vivo* biomarkers can enhance patient selection in clinical trials allowing greater diagnostic accuracy, a more homogeneous trial population, and stratification by co-pathology to create subgroups most likely to derive therapeutic benefit from DMTs.

## INTRODUCTION

Dementia with Lewy bodies (DLB) is a complex, heterogeneous, neurodegenerative dementia, with misfolded α-synuclein forming Lewy bodies (LB) and Lewy neurites as the neuropathological hallmark [1]. Clinical features are variable but a diagnosis of probable DLB requires the presence of dementia and two core features among recurrent visual hallucinations (VH), fluctuating cognition, REM sleep behaviour disorder (RBD) and parkinsonism. Currently there are no disease modifying therapies (DMTs) available in DLB and to date there have been relatively few clinical trials, mostly repurposed from AD or other conditions [2–5]. In total, 25 agents have been investigated across 40 clinical trials: 7 in phase 3, 31 in phase 2, and 2 in phase 1 [6^▪^]. There has been a recent acceleration in the focus on drug development in DLB with a recent review suggesting almost one quarter of all clinical trials in DLB are currently active (*n* = 9) [6^▪^]. Clinical trials developing DMTs are also becoming more common with over half of trials for these agents currently active in DLB [6^▪^].

The marked clinical and neuropathological heterogeneity in DLB (discussed in more detail below) presents challenges for both the diagnosis of DLB and the selection of a homogenous patient population in clinical trials. Coincident neuropathology contributing to the clinical phenotype is highly prevalent and needs to be considered in clinical trial design, particularly with the advent of therapies with specific pathological targets [1]. The use of biomarkers to identify co-pathology in DLB presents an opportunity to apply a precision medicine approach to clinical trials for DMTs with different targets allowing stratification of patients most likely to derive therapeutic benefit. In this review, we discuss the recent advances in biomarker development in DLB, and, in the context of the challenges outlined, we highlight ways in which biomarkers have the potential to transform clinical trials in DLB. 

**Box 1:**
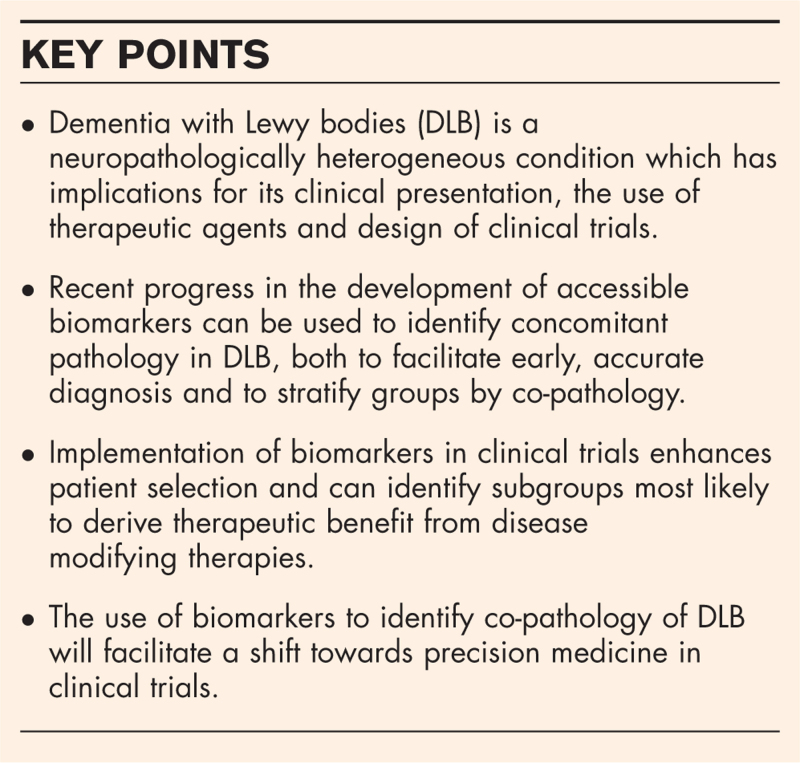
no caption available

## CHALLENGES IN CLINICAL TRIALS

### Neuropathological heterogeneity

α-Synuclein is the neuropathological hallmark of Lewy body disorders (LBD) and drives cognitive decline as it spreads neocortically [7,8^▪^]. DLB is also neuropathologically heterogeneous and up to seven pathologies can coexist creating a diverse mix of combinations with variable clinical consequences [9^▪▪^]. The few “pure” LB pathology cases usually show a more typical DLB profile with higher frequency of core features such as RBD, VH and parkinsonism [10,11]. However, the vast majority of DLB cases are “mixed”, most commonly with concomitant Alzheimer's disease neuropathological changes (ADNC), followed by other co-pathologies such as TDP-43 and cerebrovascular disease (CVD), which increase in frequency with age [9^▪▪^,^11^–^19^]. See Table 1 for a summary of the pathological changes in DLB. These co-pathologies may have synergistic effects, occurring more frequently and with greater severity together, although this needs to be delineated further [20–22]. The added presence of ADNC in LBD appears to be a particular driver for the accumulation of TDP-43, CVD and inflammatory changes in LBD [13,21,22,23^▪^,^24^]. Moreover, concomitant neuropathologies cumulatively influence the clinical phenotype, cognitive decline, and disease progression in DLB [12,18,25,26].

**Table 1:** Pathological changes in DLB with respective biomarkers.

Pathological changes	Frequency in DLB	Influence on clinical phenotype in DLB		
		Clinical presentation	Prognosis	Biomarker
Amyloid-β plaques	Aβ positivity in DLB approximately 60% [82,83,137]	Associated with increased cognitive dysfunction [81,138,139]	Rapid cognitive decline in Aβ positive individuals [81,87,139]	Plasma Aβ42/40, ptau (detects amyloid and tau) [86^▪^,^95^] CSF Aβ42 Amyloid PET
Tau neurofibrillary tangles	∼2/3 DLB with NFT Braak stage >/= III CSF p-tau elevated in 28% of DLB [31]	Higher tau burden associated with reduced parkinsonism, VH and RBD. [31,138] Inconsistently associated with increased cognitive impairment [29,31]	Associated with shorter time to dementia and shortened overall survival. [20] Tau associated greatest neuropathological index of late life cognitive decline [25].	Plasma p-tau [96,97,98^▪▪^,^99^,^101^] CSF Tau PET
α-Synuclein	Pathological hallmark of DLB.	LB pathology is a significant driver of cognitive dysfunction in DLB [7,8^▪^]. Pure LB pathology associated VH, fluctuations and core symptoms of DLB [11].	Greater cognitive decline as the pathology is more widespread and extends neocortically [7,8^▪^].	CSF SAA [66–70] Skin, olfactory mucosa, gastrointenstinal mucosa and submandibular gland SAA [59–66]
TDP-43 (LATE)	13–60% individuals [11–15]. More prevalent in advanced neocortical LB pathology and with AD co-pathology.	Degree of impairment proportional to burden of TDP-43 [23^▪^,^34^].	Rate of cognitive decline associated with burden of LATE [23^▪^,^34^].	RT-QUIC being developed, not yet in use and has not been investigated in DLB [110].
Cerebrovascular disease (CVD)	Inconsistent reports but most likely increased CVD in DLB, particularly for CAA and WMH [35,37,140]. Increases associated with AD co-pathology.	May be associated with greater cognitive impairment but studies need replication [35,141,142].	Most vascular indices associated with faster cognitive decline in late life [25].	MRI
Inflammation	Neuroinflammation occurs in early stages of DLB and decreases as the disease progresses. Inflammation less prominent than in AD. Both α-synuclein and ADNC implicated as instigators [24,143–146]	Inconsistent findings. Cognitive impairment and neuropsychiatric symptoms associated with ↑ serum IL-6, TNFα. Other studies have found no association with clinical features or ↓ plasma cytokines associated with cognitive impairment [144,146–148].	Inflammation appears to decrease in later stage disease as cognition declines. Proinflammatory profile of peripheral cytokines predicts greater progression in PD [149]. Post-mortem studies do not show microglial activation [143,150–152].	CSF Plasma TSPO PET
Synaptic dysfunction	Presynaptic accumulation of α-synuclein driving synaptic dysfunction instrinsic to synucleinopathies [114,153]. Widespread reduction in synaptic density found with PET imaging [154,155]. Some markers of synaptic plasticity (e.g. neurogranin and GAP-43) may correlate with ADNC [114,115].	Loss of synaptic density correlates with cognitive impairment [154]. Significant associations between reduced levels of synaptic proteins and cognitive impairment but not motor symptoms in DLB and PDD [115,156]. A number of other studies have found no correlation with individual synaptic proteins and cognition or PD scales in DLB [112].	↓ levels of markers of synaptic dysfunction associate with increased rate of cognitive decline in DLB [114,115,150].	PET imaging of synaptic density CSF markers include VGF, NPTXR, NPTX2, PDYN, SCG2, SNAP-25, Neurogranin and GAP-43 [114,115] Extracellular vesicles

Aβ, amyloid β; ADNC, Alzhiemer's disease neuropathological change; CAA, cerebral amyloid angiopathy; CSF, cerebrospinal fluid; GAP, growth associated protein; IL, interleukin; LATE, limbic-predominant age-related TDP-43; MRI, magnetic resonance imaging; NFT, neurofibrillary tangles; NPTXR, neuronal pentraxin; PDYN, prodynorphin; PET, positron emission tomography; p-tau; phosphorylated tau; RBD, rapid eye movement sleep behavior disorder; RT-QUIC, Real-time Quaking Induced Conversion; SAA, seeding amplification assay; SCG, secretogranin; SNAP, synaptosomal-associated protein; TNF, tumour necrosis factor; TSPO, translocator protein; WMH, white matter hyperintensities.

ADNC occurs in approximately 50% of cases with LBD and appears to have the greatest influence on clinical expression in DLB. Patients with AD co-pathology have more severe cognitive impairment, rapid cognitive decline, greater burden of neuropsychiatric symptoms and earlier mortality [20,27–31]. Both amyloid and tau have been implicated and independently influence the clinical phenotype [29,31,32]. High burden of neurofibrillary tau tangles (NFT) in DLB is associated with lower prevalence of core features and lower likelihood of DLB clinical diagnosis [31]. Some studies have found associations between tau pathology and cognitive dysfunction in DLB, with both impaired cognitive performance and shorter time to develop dementia, but these results have not been consistently replicated [20,29,31,33]. The high correlation between tau and amyloid pathologies make separation of their respective influences challenging and may underlie some of the differences found between studies.

Recent evidence suggests that the burden of limbic-predominant age-related TDP-43 encephalopathy (LATE) contributes additively to cognitive dysfunction in DLB [23^▪^,^34^]. CVD, an umbrella term to include cerebral amyloid angiopathy (CAA), infarcts, microbleeds and white matter hyperintensities (WMH), is common in DLB [1,35,36]. Most studies support an increased prevalence of CVD in DLB, particularly for CAA and MRI-detected WMH, but it is unclear whether this is an independent association or linked to AD co-pathology [35,37]. One in-vivo study suggested that although CVD and ADNC co-existed in DLB, their contributions to neurodegeneration appeared to be region specific [8^▪^]. Other studies have indicated reduced LB burden in the context of CVD, possibly due to a lowering of the threshold for dementia [29,38]. There is not yet consensus as to the clinical impact of CVD; WMH and CAA have been associated with greater cognitive impairment in DLB but this needs replication, particularly given these pathologies also associate with ADNC [35].

A number of recent studies have searched for endophenotypes within DLB to delineate the influence of the various neuropathologies [39,40]. Disentangling the influence of respective neuropathies to allow a precision medicine approach is of increasing importance with the advent of DMTs aiming to target specific neuropathological changes. Identification of DLB subgroups with AD or neuroinflammatory pathological changes will be of particular value given these are targets of a number of novel DMTs in AD and PD [41,42].

### Patient selection

Accurate diagnosis of DLB is a challenge in clinical and research settings, both due to the clinical overlap with other dementias and the lack of easily available biomarkers. Underdiagnosis is common with wide discrepancy between the frequency of LBD in post-mortem samples (15–20%) and clinical prevalence (5%) [43–47]. Presence of concomitant pathologies such as ADNC and TDP-43 contribute to the diagnostic inaccuracy [48,49]. Additionally, the clinical heterogeneity results in patients being evaluated by different specialties (psychiatry, movement disorders, sleep, memory clinics, among others) depending on the primary symptom and referral pathways [50]. Thus the characteristics of the cohort recruited in clinical trials will depend on the setting and recruitment method which is often not reported and may vary widely.

#### Lewy body disorders spectrum

LBD encompasses the diagnostic spectrum from Parkinson's disease (PD) to DLB, and while Parkinson's disease dementia (PDD) and DLB have many neuropathological, clinical and genetic commonalities, there is also evidence to support distinctions. There is greater clinicopathological overlap with AD in DLB than in PDD [51] which may account for the difference in treatment response to a number of commonly used drugs across DLB and PDD [52–54]. Indeed, the current diagnostic criteria support the continued use of the ‘one year rule’ to distinguish PDD and DLB by the chronicity of their cognitive impairment relative to motor symptoms [11].

Despite these group differences there is no single clinical or neuropathological feature which can uniquely differentiate PDD and DLB which presents a challenge for clinical trials. This is reflected in the recruitment to clinical trials for DLB which lacks uniformity and often includes additional diagnostic groups, usually PDD (Table 2). Recent expert consensus suggest that DLB and PDD could be included in clinical trials as separate, adequately powered groups, to allow identification of group differences [55]. Increasingly, clinical trials for DMTs are restricting their patient populations to DLB alone which likely reflects efforts to create a more homogenous group with less diverse pathomechanisms [6^▪^]. The development of biomarkers to differentiate underlying biological differences between PDD and DLB from would be of great use here.

**Table 2:** Patient population, stage of dementia and use of biomarkers in clinical trials of DLB active from 2017 to present.

Agent	Phase	Trial duration	Therapeutic purpose	Patient population	Stage of dementia	Use of biomarkers	
						Inclusion	Outcome
Ambroxol	1	November 2023– November 2025	DMT	DLB	Mild/moderate dementia	CSF (tau, p-tau and beta amyloid 42) DaT scan	Plasma (α-synuclein, tau, p-tau, beta amyloid-42) CSF (α-synuclein, tau, p-tau, beta amyloid-42) MRI (hippocampal and ventricular volume)
CT1812	2	June 2022– April 2024	DMT	DLB	Mild/mod dementia	MRI	CSF and plasma (α-synuclein)
Memantine	3	March 2022– April 2025	Symptomatic	PDD+DLB	All dementia stages		
Fosgonimeton (ATH-1017)	2	January 2022– November 2023	DMT	PDD+DLB	Mild/mod dementia		
Ondansetron	2	October 2021– January 2025	Symptomatic	PD+DLB	All dementia stages		
Terazosin	1	October 2021– October 2022	DMT	DLB	Mild/moderate dementia		MRS FDG-PET Serum ATP
CST-103, CST-107	2	June 2021– August 2022	Symptomatic	PDD/RBD+ DLB/MCI/PD	Mild/mod dementia		Digital wearable device
Ambroxol	2	May 2021– December 2023	DMT	DLB	Prodromal/mild dementia	DaTSCAN ECG qEEG, CSF (amyloid-beta) Plasma (APOE-4, GBA)	DaTSCAN qEEG ECG MRI CSF and plasma (tau, p-tau and beta amyloid 42, GCase, α-synuclein)
Irsenontrine	2	February 2021– January 2022	Symptomatic	PDD+DLB	Mild/mod dementia	CSF and plasma (cGMP, ttau, Aß42/40, GFAP, neurogranin, ptau181, NfL, APOE4)	CSF and plasma (cGMP, ttau, Aβ42/Aβ40, GFAP, neurogranin, ptau181, NfL, APOE4)
Zonisamide	2	February 2021	Symptomatic	PDD+DLB	MCI/prodromal	PSG MIGB DaTSCAN	MIGB DaTSCAN
Zonisamide	2	September 2020	Symptomatic	PDD+DLB	Mild/mod dementia		
NYX-458	2	November 2019– December 2022	Symptomatic	PDD+DLB	Prodromal/mild dementia		
Neflamapimod	2	September 2019– June 2020	DMT	DLB	Mild/mod dementia	DaTSCAN (if negative PSG confirmation of RBD)	qEEG Plasma Aß42, ptau181
Nilotinib	2	July 2019– April 2023	DMT	DLB	Mild/mod dementia	DaTSCAN	Florbetaben PET (amyloid) CSF (HVA) Plasma
HTL0018318	2	July 2019– September 2019	Symptomatic	DLB		SPECT or MIBG	
Bosutinib	2	April 2019– April 2021	DMT	DLB	Mild/mod dementia	DaTSCAN	Plasma and CSF (HVA, DOPAC, Abeta40/42, total tau, ptau231/181 and total and oligomeric alpha-synuclein). Inflammatory markers including triggering receptors on myeloid cells (TREM)-2 CSF markers of cell death including neuron specific enolase (NSE), S100B and phosphorylated neurofilaments.
Irsenontrine	2	May 2018– April 2020	Symptomatic	DLB	Mild/mod dementia		
LY3154207 (Mevidalen)	2	November 2017– July 2020	Symptomatic	PDD+DLB	Mild/mod dementia		Actigraphy, Lilly trial app
Zonisamide	3	October 2017– December 2020	Symptomatic	DLB	Mild/mod dementia	WBC platelet Serum AST ALT ALP GTP	
Pimavanserin	3	September 2017– October 2019	Symptomatic	All cause dementia	All dementia stages		
Intepirdine	2	October 2016– November 2017	Symptomatic	AD+PDD+DLB	Mild/mod dementia		Electronic walkway assessment, mini balance evaluation systems test
Vodobatinib (K0706)	2	September 2016– October 2023	DMT	DLB	Mild/mod dementia	ECG	Plasma and CSF (HVA, DOPAC, Abeta40/42, t-tau, ptau231/181 and total and oligomeric alpha-synuclein)
Nelotanserin	2	March 2016– May 2018	Symptomatic	PDD+DLB	Mild/mod dementia		Video-PSG Actigraphy
Intepirdine	2	January 2016– December 2017	Symptomatic	DLB	Mild/mod dementia		
Nelotanserin	2	December 2015– November 2017	Symptomatic	PDD+DLB	Mild/mod dementia		
Zonisamide	3	April 2015– November 2017	Symptomatic	DLB	All dementia stages		

Aβ, amyloid-β; AD, Alzheimer's disease; APOE, apolipoprotein E; ATP, adenosine triphosphate; CSF, cerebrospinal fluid; cGMP, cyclic guanosine monophosphate; DaTSCAN, dopamine transporter single photon emission computerized tomography; DOPAC, 3,4-dihydroxyphenylacetic acid; ECG, electrocardiogram; FDG-PET, fluodeoxiglucose positron emission tomography; GBA, glucosylceramidase β; HVA, homovanillic acid; MIBG, iodine-123 metaiodobenzylguanidine myocardial scintigraphy; MRI, magnetic resonance imaging; NSE, neuron-specific enolase; PET, positron emission tomography; PSG, polysomnography; qEEG, quantitative electroencephalogram; S100B, S100 calcium-binding protein B; SPECT, single photon emission computerised tomography; TREM-2, triggering receptor expressed on myeloid cells 2; WBC, white blood cell.

#### Prodromal dementia with Lewy bodies

To date, most clinical trials in DLB have included patients in the mild to moderate dementia stages, but there has been a recent shift towards including patients at the prodromal stage [6^▪^] (Table 2). Enrolling patients in the prodromal stages is critical for early intervention with symptomatic treatments or DMTs while the pathological burden is limited and before clinical symptoms become advanced. Publication of research criteria for the diagnosis of mild cognitive impairment with Lewy bodies (MCI-LB) has provided a framework for recruitment that has facilitated this transition and these criteria have recently been validated [56,57^▪▪^]. Isolated delirium and psychiatric-onset prodromal presentations of DLB are recognised but have not yet been fully characterised [56]. Accurate diagnostic biomarkers are needed to identify the broad spectrum of clinical presentations in the prodromal stages of DLB and differentiate the influence of co-pathologies at this stage [58].

## USE OF BIOMARKERS IN DEMENTIA WITH LEWY BODIES

### Diagnostic biomarkers in dementia with Lewy bodies

Current indicative biomarkers for the clinical diagnosis of ‘probable’ or ‘possible’ DLB include dopamine transporter (DAT) scan, myocardial scintigraphy and polysomnography to confirm RBD [11]. Additional supportive biomarkers are not used in diagnosis but can strengthen the overall diagnostic evaluation [11]. Although the biomarkers currently used in the diagnosis of LBD do not provide direct evidence of Lewy-related pathology, recent promising results have been reported for a seeding amplification assay (SAA) that detects α-synuclein in cerebrospinal fluid (CSF), blood, skin, olfactory and gastrointestinal mucosa in addition to other tissues [59–70]. The CSF α-synuclein real-time quaking-induced conversion (RT-QuIC) assay identified clinicopathologically confirmed PD and DLB with a sensitivity and specificity of >90% [66–71]. CSF α-syn RT-QuIC also appears to be a robust biomarker in prodromal DLB in both MCI and isolated RBD presentations [72^▪▪^,^73^,^74^]. A recent meta-analysis indicated the diagnostic accuracy was highest in skin and CSF αSyn SAA with specificity highest in pathologically confirmed diagnoses [72^▪▪^]. However, although this assay offers potential as a diagnostic tool for both clinical and research purposes, currently inter- and intra- laboratory variations remain high, and more information about the longitudinal course and quantitative values rather than a dichotomous result are needed [66]. Immunoassays also find higher blood levels of phosphorylated α-synuclein in PD patients but levels in DLB patients, and the association with CSF α-synuclein RT-QuIC measurements, remain largely unknown and warrant future studies [75–77].

### Biomarkers for co-pathology

Disease-specific biomarkers are needed to identify each co-pathology in-vivo and delineate their contribution in DLB. Biomarkers reflecting AD pathophysiology are well characterised and until recently principally included PET scans and CSF [78]. These biomarkers have been studied in DLB; up to 22% of DLB cases have an AD CSF biomarker profile [79] and PET imaging suggests 59% of individuals with DLB have β-amyloid deposition (Aβ+) [80–83]. Importantly, an AD CSF profile, PET-amyloid and PET-tau burden have been associated with cognitive decline in DLB [29,33,84,85,86^▪^,^87^], Recently, highly promising plasma Aβ and phosphorylated tau (p-tau) assays have been developed, offering less invasive and more accessible blood-based biomarkers [88]. In particular, plasma measures of p-tau at threonine 181, 217 and 231 (p-tau181, p-tau217, p-tau231) show comparable diagnostic accuracy to CSF and PET biomarkers for AD [89–93]. Plasma p-tau181 is closely related to ADNC, reflecting both tau and early amyloid deposition, and is most discriminatory in pathologically confirmed AD, irrespective of clinical presentation [94–96].

There is early evidence that these plasma biomarkers may also have clinical utility in DLB. Post-mortem studies show significantly higher plasma p-tau181 and p-tau231 in patients with concomitant AD pathology over LB alone; p-tau181 appears to be highly specific and predictive of AD pathology irrespective of co-pathology [96,97]. Additionally, plasma p-tau181 correlates with AD CSF biomarkers, PET-tau and PET-amyloid imaging in DLB; and levels of plasma p-tau181 are higher than controls in both probable DLB and MCI-DLB [86^▪^,^98^^▪▪^,^99^,100^▪^]. These results suggest that plasma ptau181 could also be used as a marker of AD co-pathology in DLB [86^▪^,100^▪^]. Moreover, in DLB in patients with pathological CSF Aβ, elevated plasma ptau-181 and ptau217 are associated with longitudinal cognitive decline, likely reflective of the ADNC contribution [98^▪▪^,^99^,^101^]. No difference in plasma p-tau-181 levels was found between LBD patients with or without a positive amyloid PET scan in one large study and a second smaller post-mortem study [102,103^▪^]. However, subgroup analysis of patients in the first study did demonstrate the discriminant ability of plasma biomarkers in detecting Aβ+ and Aβ− cases, such that the negative finding in the earlier study may reflect the inclusion of both PDD and DLB patients and the combination of different sites with use of different imaging ligands and procedures [86^▪^,^102^]. Regardless, it is important to consider that strategies specific to LBD may be needed to detect concomitant AD pathology [104,105].

In-vivo detection of AD co-pathology in DLB can give valuable prognostic information and may become useful in the design of clinical trials, particularly to identify participants for DMTs targeting specific neuropathologies such as Aβ. Increasing evidence suggests that the relationship between Aβ and plasma biomarkers occurs irrespective of overarching diagnosis and it seems likely that combination of plasma biomarkers will have the highest accuracy in identifying AD co-pathology in LBD [86^▪^]. However, before these blood-based biomarkers can be incorporated into research and clinical practice in LBD, further validation is needed with standardisation of assays and establishing appropriate cut-offs to ensure robustness and reliability in diverse cohorts [106].

Besides LB and AD pathology, neuroimaging and biofluid markers related to other disease mechanisms such as CVD, neurodegeneration, synaptic loss and neuroinflammation have been reported in LBD [24,35,107–109]. Efforts are currently underway to develop a biomarker for TDP-43 and one recent study reported some success with a CSF assay but this has not been validated in DLB [110]. Neurofilament light (NfL) is a disease non-specific marker of axonal degeneration which is elevated in the plasma and CSF in DLB from the early prodromal stages and is associated with increased cognitive decline [111^▪^,^112^]. Similarly, GFAP is also elevated in DLB and evidence suggests it is a marker of amyloid pathology, showing preferential increases in post-mortem and CSF Aβ+ individuals with DLB, and it has been associated with lower MMSE scores [101,103^▪^,^113^]. There are also promising candidate biomarkers of synapse dysfunction in DLB, particularly those related to neurotransmitter transport and secretion synapse such as VGF, PDYN, SCG2 and neuronal pentraxins (NPTX) which enhance diagnostic accuracy when combined with existing biomarkers [114,115]. Isolating neuronal-derived exosomes from plasma is another promising approach that allows identification of synaptic proteins without requiring CSF [108]. Non-specific biomarkers can be confounded by comorbid neuropathology and they may be best used in combination with other markers or in a prognostic capacity. For example, combination of multiple plasma biomarkers (Aβ42/40, neurofilament light, glial fibrillary acidic protein (GFAP) and p-tau181) has shown to improve accuracy of identifying Aβ+ cases in DLB [86^▪^].

However, relative to AD, there is a dearth of research in the exploration of biomarkers in LBD and much of the existing data are limited by factors such as small sample size, lack of longitudinal studies and reproducible findings. Greater efforts are needed to profile biomarkers with various pathophysiological mechanisms in DLB in large collaborative longitudinal studies. Machine learning techniques and emerging high-throughput proteomic profiling platforms such as Olink and SomaScan may also prove useful for identifying co-pathologies and discovering novel mechanistic pathways implicated in LBD [116,117].

### Biomarkers in clinical trials

Given the challenges in DLB drug development, advances in biomarkers offer a significant opportunity to improve clinical trials. Biomarkers can be classified according to their context of use [118]; Table 3. Diagnostic biomarkers confirm the presence of disease and can be used to enhance patient selection, allowing greater homogeneity and addressing the diagnostic difficulties with DLB. Predictive biomarkers identify individuals most likely to respond to intervention, facilitating group stratification into those most likely to derive therapeutic benefit. This is particularly relevant in DLB given the inherent neuropathological heterogeneity; selection of participants with specific biomarker signatures will optimise clinical trials for DMTs aiming to target specific neuropathologies. For example, anti-amyloid DMTs approved in AD may have efficacy in patients with DLB where there is evidence of Aβ deposition.

**Table 3:** The Food and Drug Administration (FDA) Biomarkers, EndpointS, and other Tools (BEST) classification [117]

Biomarker	Definition
Risk/susceptibility	Indicates potential for developing the disease in an individual without clinical signs of the disease
Diagnostic biomarker	Detects or confirms the presence of disease or condition
Monitoring	Serial measurement to assess status of disease to detect effect or find evidence of exposure to the therapeutic agent
Pharmacodynamic/response	Changes in response to exposure to agent
Predictive biomarker	Presence or change in the biomarker predicts an individual or group more likely to experience a favourable/unfavourable effect from exposure to agent
Prognostic biomarker	Identifies likelihood of clinical event, disease recurrence or progression
Safety	Measured before/after exposure to agent to indicate likelihood, presence or extent of toxicity as adverse event

Enrichment with biomarkers can also improve statistical power and reduce required numbers in a patient population with considerable recruitment challenges [119,120]. Indeed, biomarkers are an integral part of AD clinical trials and have been used effectively to reduce the sample size required to show clinical benefit and identify subgroups of patients most likely to benefit from DMTs such as donanemab [121,122]. Although CSF and PET have traditionally been used to screen eligible participants, recent trials have preferentially included plasma biomarkers as a more cost effective and accessible alternative [106].

To date, although more than half of DLB clinical trials have included biomarkers, these are more commonly used as outcome measures than to support inclusion criteria Table 2. While DAT scans have been used as diagnostic biomarkers in some trials, this also has the potential to select more parkinsonian phenotypes of LBD, and DAT scans may be negative in autopsy confirmed DLB where there is little nigral neurodegeneration or brainstem involvement [11,123]. Indeed, recent post-mortem work has suggested two distinct pathways of progression in LBD: body-first with caudo-rostral progression or brain-first with initially amygdala-centred involvement following a caudal progression [124,125].

Given the clinical implications of AD co-pathology and possible impact on treatment outcomes, the inclusion of amyloid-β and tau biomarkers in LBD clinical trials have the potential to stratify accordingly and select subgroups most likely to benefit as well as confirming target engagement in DMTs. In fact, amyloid-β positive individuals with DLB were found to have greater response to acetylcholinesterase inhibitors than amyloid-β negative individuals [126]. The use of AD biomarkers as predictive biomarkers to stratify treatment response is increasing in clinical trials for DLB and have been included in recent trials for irsenotrine, neflamapimod and ambroxol [127–129,130^▪^] (Table 2). For irsenotrine no differential treatment outcomes were found according to amyloid status [127] but in the phase 2 clinical trial for neflamapimod a greater magnitude of effect was found in patients with DLB without evidence of AD co-pathology [130^▪^,^131^]. These early applications are promising and the use of biomarkers to stratify clinical trials is likely to continue in the future. There is an active drug development pipeline in AD with 172 clinical trials ongoing and DMTs representing over 80% of agents [42]. Identifying DLB patients with AD co-pathology has the potential to select patients who may benefit most from repurposed medications aiming to target amyloid or tau proteinopathies. Co-pathologies may also influence treatment responses to DMTs aiming to target LB pathology directly and thus stratifying patient groups based on biomarker defined co-pathology may be of importance regardless of the treatment target.

Biomarkers for α-synuclein, as described above, are also needed in LBD trials and would be of particular use as diagnostic biomarkers for recruiting participants in the prodromal or preclinical stages. Clinical trials are ongoing for tyrosine kinase inhibitors and CT1812, which in preclinical studies increased clearance of α-synuclein, amyloid and hyperphosphorylated tau [132]. In these clinical trials, plasma and CSF biomarkers have been included as outcome measures to assess target engagement and delineate the effect on comorbid pathologies. Indeed, a recent trial of nilotinib in PD failed and it was found not to penetrate the CNS because there was no change in CSF dopamine metabolites [133]. Novel biomarkers such as neuronal-derived exosomes also have potential as measures of target engagement for DMTs affecting neuronal pathways [134]. Although the therapeutic effect of these agents in DLB has not yet been reported, given the dual action, stratification by co-pathology may be a useful strategy.

## CONCLUSION: MOVING TOWARDS A BIOLOGICAL DEFINITION OF LEWY BODY DISORDERS

It is now well established that the clinical presentation historically applied to define probable AD was often not reflective of the underlying neuropathology [135]. The same diagnostic difficulties are apparent in DLB with comorbid neuropathologies contributing further to reduce diagnostic accuracy [48,49]. A biological definition is needed both to clearly define DLB and to delineate the contribution of co-pathologies, and support for this is growing in PD [136]. Recent approval of anti-amyloid therapies in AD accentuate the need for a biological diagnosis for DLB, particularly given up to 50% of patients with DLB have AD co-pathology [42,136]. Biomarkers will be critical in supporting early diagnosis and identifying a patient-specific neuropathological footprint to facilitate a shift towards precision medicine with stratification of subgroups most likely to benefit from DMTs in clinical trials.

## Acknowledgements

*None.*

### Financial support and sponsorship

*L.L.G. is funded for a PhD by the Alzheimer's Society. C.A. postdoctoral fellowship is funded by the Susan and Charles Berghoff Foundation.*

### Conflicts of interest

*C.A. has received the Sue Berghoff LBD Research Fellowship, and honoraria as speaker from F. Hoffmann-La Roche Ltd, Zambon, Nutricia, Schwabe Farma Ibérica S.A.U. She is member of the Board of Directors of the Lewy Body Dementia Association. L.L.G., J.C. and C.B. have no conflicts of interest. DA has received research support and/or honoraria from Astra-Zeneca, H. Lundbeck, Novartis Pharmaceuticals, Evonik, and GE Health and has served as paid consultant for H. Lundbeck, Eisai, Heptares, Mentis Cura, Eli Lilly, and Biogen.*

## REFERENCES AND RECOMMENDED READING

Papers of particular interest, published within the annual period of review, have been highlighted as:

▪ of special interest
▪▪ of outstanding interest
